# Towards a simultaneously speaking bilingual robot: Primary study on the effect of gender and pitch of the robot’s voice

**DOI:** 10.1371/journal.pone.0278852

**Published:** 2022-12-28

**Authors:** Hamed Pourfannan, Hamed Mahzoon, Yuichihiro Yoshikawa, Hiroshi Ishiguro

**Affiliations:** 1 Intelligent Robotics Laboratory (Hiroshi Ishiguro’s Laboratory), Department of Systems Innovation, Graduate School of Engineering Science, Osaka University, Osaka, Japan; 2 Institute for Open and Transdisciplinary Research Initiatives (OTRI), Osaka University, Osaka, Japan; Vinnytsia National Technical University, UKRAINE

## Abstract

With fast and reliable international transportation, more people with different language backgrounds can interact now. As a result, the need for communicative agents fluent in several languages to assist those people is highlighted. The high cost of hiring human attendants fluent in several languages makes using social robots a more affordable alternative in international gatherings. A social robot capable of presenting a piece of information in more than one language at the same time to its audience is the goal of this line of study. However, the negative effect of background noise on speech comprehension in humans is well-established. Hence, presenting a piece of information in two different languages at the same time by the robot creates an adverse listening condition for both individuals listening to the speech of such a bilingual robot. In this study, we investigated whether manipulating the pitch and gender of the robot’s voice could affect human subjects’ memory of the presented information in the presence of background noise. The results indicate that the pitch and gender of the speaking voice do indeed affect our memory of the presented information. when a male voice was used, a higher pitch resulted in significantly better memory performance than a lower pitch. Contrarily, when a female voice was used, a lower pitch resulted in significantly better memory in participants than a higher pitch. Both male and female subjects performed significantly better with a female voice in a noisy background. In nutshell, the result of this study suggests using a female voice for robots in noisy conditions, as in the case of simultaneously speaking robots, can significantly improve the retrieval of presented information in human subjects.

## 1 Introduction

With fading borders between countries thanks to fast and reliable transportation, the number of situations where people from different language backgrounds interact with each other is increasing. When people with different languages gather in the same place, it follows the need to convey the information they need in their own preferred language. Thus far, general procedures such as public address (PA) systems have been used to broadcast important information in several different languages in places such as airports and exhibitions. However, this is far from a one-to-one personalized interaction that would make a deeper connection and convey information more efficiently while creating a more friendly atmosphere. Particularly, if persuasion or product recommendation is the final goal in mind, research shows that one-to-one interaction would significantly increase the chances of success [1]. Considering the scarcity and cost of recruiting human personnel fluent in several languages, and their availability 24 hours whenever needed, a robot capable of doing the same is more cost and time-efficient in a crowded international environment.

Robots capable of speaking several languages are not new. They have already been used as translators for tourists in Japan [2, 3]. As well as Wakamaru, a social robot developed by Mitsubishi Heavy Industries that can greet people in the office and hotels in several different languages [4]. However, such multilingual robots are not intended to be used to present information in more than one language simultaneously. Currently, each person should wait for the other user to finish their inquiry to the robot before he/she can make a new inquiry in their own preferred language. This line of study aims to update the status quo by introducing the concept of social robots that speak two languages at the same time.

A simultaneously speaking bilingual robot is a social robot with the same speech synthesis and speech recognition systems that would be implemented in a normal robot but with one main difference, it expresses the same speech content in two languages at the same time, so that two groups of people with different language backgrounds can understand the speech presented by the robot instantly. One of the challenges that need to be considered in designing such a simultaneously speaking bilingual robot is the well-documented negative effect of background noise on speech comprehension in general. This negative effect is shown to be stronger when speech is used as the background noise [5–9]. As a result, the described scenario where the robot creates utterances in two languages simultaneously creates an adverse listening condition for both the users of such a robot. Several factors can potentially affect the speech comprehension of human subjects listening to a bilingual robot. In this study, we focus on the voice characteristics of such a robot. More specifically, the question we tried to answer in this study was whether using a specific voice gender, and voice pitch could improve speech comprehension in human subjects listening to the robot while it speaks in two different languages simultaneously.

## 2 Present study

This study consists of two experiments in which we investigated the effect of different combinations of voice-gender and voice-pitch on the performance of human subjects in different memory tasks in monolingual (Experiment 1) and bilingual contexts (Experiment 2). The existing literature on the effect of voice gender and voice pitch on speech comprehension is controversial. No decisive information exists regarding which voice gender and voice pitch can potentially result in better speech comprehension and memory in noise. Therefore, in this study, we investigate this topic more in-depth.

### 2.1 Voice gender

Noyes and Frankish suggested that content presented by a female voice is more difficult to recognize [10]. A later functional Magnetic Resonance Imaging (fMRI) study proposed that comprehension of a piece of information presented by a female voice requires more verbal processing compared to when the same information is presented by a male voice. This slower verbal processing of the female voice is demonstrated in a slower response time and increased activity in the auditory cortex of the human brain when listening to a female voice [11]. More recent work supports the idea that understanding the content presented by a female voice might require more processing than a male voice by suggesting that when listening to a male voice, brain regions responsible for “what was said” is more active contrary to a female voice which activates the parts of the brain responsible for “how and by whom it was said” which might be responsible for the delayed response time when listening to a female voice [12]. However, later works could not confirm the results obtained in earlier studies and suggested that voice gender does not significantly affect the verbal performance of participants [13].

Conversely, other studies observed faster reaction times when using a female voice, suggesting that these gender effects might play a role in the early stages of phonological processing and that their effect dissolves before the word meaning is processed [14, 15]. This is in accordance with the other study investigating the effect of voice gender, along with other factors on the speech intelligibility of participants in a verbal disaster warning system. Their result suggests that although the gender of the voice does not affect speech intelligibility significantly, using a female voice increases participants’ sense of urgency and can result in a faster evacuation [16]. In another experiment that considered the subjective evaluation of participants about the presented voice gender, researchers found a significant preference towards a female voice [17]. Almost all digital assistants of famous companies use a female voice on their devices by default. The reason for this choice of voice gender is suggested to be due to their customers’ more positive feedback toward using a female voice as users find a female voice “more trustworthy and supportive [18]”. This has been supported by other independent surveys finding that younger participants prefer a female voice over a male voice in general [19].

### 2.2 Voice pitch

The pitch level is determined by the fundamental frequency of a given voice measured in hertz (Hz). The pitch is averaged at approximately 120 Hz for males, and 210 Hz for females [20]. Voice pitch has been shown to have a significant effect on people’s ratings of another person’s attractiveness. One study suggests that male subjects prefer a high-pitch female voice over a low-pitch female voice, while female subjects prefer a low-pitch male voice over a high-pitch male voice [21]. In another study, female subjects preferred a high-pitch voice to a low-pitch voice when the talker was female [22]. Contrary to the above-mentioned studies, however, other studies suggest an opposite pattern of findings. In one study, the low-pitch voice led to a more positive rating by participants over a high-pitched voice in general [23]. Furthermore, another study suggests that a high-pitch male voice was rated higher in their study than a low-pitch male voice [24].

The results are mixed when considering the effect of voice pitch on speech intelligibility in a competing-talker scenario as well. While some works suggest that a difference between the pitch of the target talker and that of the background talker improves speech intelligibility [25, 26], other works suggest the effect of voice pitch on speech intelligibility to be smaller than previously thought [27] or find the effect stronger only in older individuals and those with a higher working memory capacity [28]. Inconsistency between the result of different studies on this matter leaves the question of what voice pitch should be used in a simultaneously speaking bilingual robot to increase the speech comprehension of subjects in noise, unanswered.

### 2.3 Research rationale

The contradictory pattern of results in the previous studies on the potential effect of voice gender and voice pitch on speech intelligibility, memory, and subjective evaluation of human subjects indicates the need for independent research to investigate this topic further. This need is more highlighted considering the existing studies on the importance of paralinguistic information, such as voice gender, voice pitch, and speaking rate, on the processing, and storage of speech information, as well as acceptance and likability toward the speaking person [13, 29, 30]. In this study, we systematically varied the gender and pitch of the speaking voice in monolingual and bilingual scenarios and recorded participants’ responses for each condition in search of any significant differences in their memory and speech comprehension scores.

## 3 Experiment 1

In this study, we examined the extent to which the pitch and gender of the speaking voice and the presence of white noise in the background, affect the listeners’ retention of the presented auditory content, demonstrated in their scores in word-list recognition, word-list immediate recall, and word-list delayed recall tasks.

### 3.1 Methods

#### 3.1.1 Participants

A total of 144 subjects (88 female, 56 male) aged between 18 and 50 years (mean = 31.6, SD = 8.0) participated in this study. As the beginning of this study coincided with the onset of the COVID-19 pandemic and limitations were imposed on on-site test conductance, we had to conduct the study online. For this purpose, we used the Prolific platform, an online research participant recruitment system. The participants were monolingual English-speaking people, currently residing in the United States of America. The invitation to participate in this study was only sent to those people who already had declared to Prolific that they do not have any hearing difficulties and have reliable headphones at their disposal. At the beginning of the experiment, participants were asked to adjust the volume of their computer while listening to the practice trial of the working memory task to make sure they hear all digits without any difficulty. The correct list of digits was shown to each subject afterward to make sure they heard it correctly.

#### 3.1.2 Design and procedure

A mixed-subject, repeated-measures design was implemented in this study. The independent variables were voice pitch (high pitch, low pitch), voice gender (male and female), participant’s gender (male and female), and presence of white noise (noise and no noise). The dependent variables were word-list recognition, word-list immediate recall, and word-list delayed recall. The participants were asked to wear headphones during the test. This study was approved and supervised by the Ethical Committee of Osaka University. At the beginning of the experiment, after a written consent form was confirmed by each participant, they underwent a backward digit span test as a rough indication of their working memory capacity. This score was used as a covariate factor to control for the effect of participants’ working memory capacity on their scores in the experiment. Based on previous studies, the working memory score of subjects can predict their ability to comprehend speech under noisy conditions [7].

The backward digit span test is a reliable task for the evaluation of working memory capacity in human subjects [31]. Usually, this test includes two blocks, and the final score is the average of the score of the participant in both blocks. However, to keep the experiment short and prevent participants’ frustration, we used only one block. Each participant listened to a list of digits starting from four digits (a 3-digit list was used as a practice trial) and going up to eight digits, resulting in five trials. After hearing each list, participants were asked to write down the same digits they heard in the list in the opposite order.

**Fig 1:**
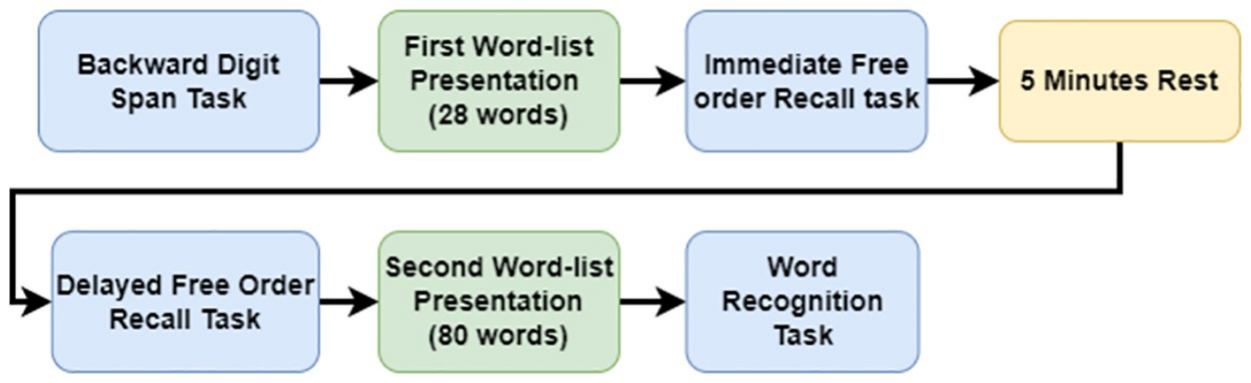
Flowchart of the experiment.

After completing the working memory section, the wordlist recall part was obtained. In the word recall task, the participants listened to a list of 20 words. One word was presented at a time with a 3-s interval. The flow of the experiment is illustrated in Fig 1.

After listening to the first wordlist (20 words), the participants were asked to write down as many words as they could remember from the list in a free order. After the responses were obtained, participants were asked to rest for 5 min and return to the test afterward. During this period, the screen was deactivated, and the participant could not proceed. After 5 min of rest, they were asked again to remember as many words from the same list as possible in a free order as a test of delayed recall ability. A simple attention check was conducted after 5 minutes of rest in between the experiment to evaluate the attentiveness of the participants during the test procedure. In this attention check, participants were asked to count down from 31 to 1 in an alternate manner (31, 29, 27, 25 …). They were then asked if the number 19 was among the numbers they counted. All the participants answered correctly and passed the attention check.

Next, the participants were asked to listen to the second wordlist, which comprised 80 words. One word was presented at a time. After the list of words was presented, participants were shown a list of 160 words one by one, 80 of which were the ones they heard in the list, and the other 80 words were new words with the same two-syllable, emotionally neutral, phonemic structure. A two-alternative forced-choice (2 AFC) design was used for the recognition task. Participants were presented with a word at a time and should decide whether they have heard the word or not by pressing “Y” or “N” on their keyboard. New and old words were mixed and presented in a manually randomized order. The experiment took about 15 minutes including 5 minutes of mandatory rest in between to prevent fatigue.

#### 3.1.3 Materials

The material used in this experiment included a list of 20 words for the recall task and 80 words for the recognition task. Each word was expressed by one of four voices: low-pitched male voice, high-pitched male voice, low-pitched female voice, and high-pitched female voice, resulting in five words for each voice condition in the recall task, and 20 words for each voice condition in the recognition task. One word from each voice condition was presented at a time, and the order of presentation was randomized such that the next word’s voice category was not predictable. The wordlists were then converted to an auditory format to be used in the experiment using the commercial version of the Notevibes AI voice generator. For the male voice, the character David was used, with an average fundamental frequency of 205 Hz, and for the female character, Lucy was used, with an average fundamental frequency of 243 Hz. Then, to adjust the pitch of the voices, the original voices were digitally edited in the open-source software “Audacity” resulting in a lower-pitched male voice with a fundamental frequency of 185 Hz, and a higher-pitched male voice with a fundamental frequency of 225 Hz. The edited female voice was set to 223 Hz for the low-pitched voice and 260 Hz for the high-pitched voice. Changing the voice pitch to more than the range of approximately 20 Hz would result in an unnatural voice; therefore, we stuck to the natural sounding range and did not modify the pitch any further.

The list of words was presented without any background noise for the no-noise condition. For the noisy condition, pure white noise (20–5000 Hz) was constantly played while reading the words for the participants with a signal-to-noise ratio (SNR)of -5 dB for the white noise. All audio files were normalized in terms of loudness to the standard -23 loudness unit full scale (LUFS) using the Audacity software. Furthermore, we checked The Long-Term Average Spectrum (LTAS) of all the clean speech excerpts before adding the white noise using Praat software to make sure that all the speech stimuli have the overall same long-term average spectrum. The final audio files generated for the experiment were two-channel stereo sounds, and the target voice and the background noise were presented from the same direction. The task was created and conducted using the online version of Psychopy software, which is a Python-based GUI-enhanced platform for designing neuroscientific and psychological experiments.

The score of subjects in the immediate word recall and delayed word recall tasks were obtained by counting the number of words remembered correctly by the subject from each voice condition. For the word recognition task, the score of subjects was obtained by counting the number of words participants could recognize correctly from the presented list of words. The score of the subjects in the recall task could range from a minimum of 0 to a maximum of 5 for each condition (twenty words in total). And, for the recognition task, the score of subjects could range from 0 to 20 for each condition (80 words in total). The final score of the subject in each task was the sum of all the words he could correctly remember from each condition. This raw data was then analyzed without any further conversion.

#### 3.1.4 Results

The effect of voice gender, voice pitch, and the presence of noise on the memory performance of the participants in the three tasks of word recognition, immediate word recall, and delayed word recall were analyzed using repeated measures ANCOVA while controlling for the potential effect of participants’ working memory capacity on their performance. The within-subject factors in the analysis were voice-gender (male or female) and voice-pitch (low -or high-pitch). The between-subject factors were the participants’ gender (male or female) and noise condition (noise or no noise). A p-value of 0.05 was used for all the statistical analyses conducted in this study.

**Fig 2:**
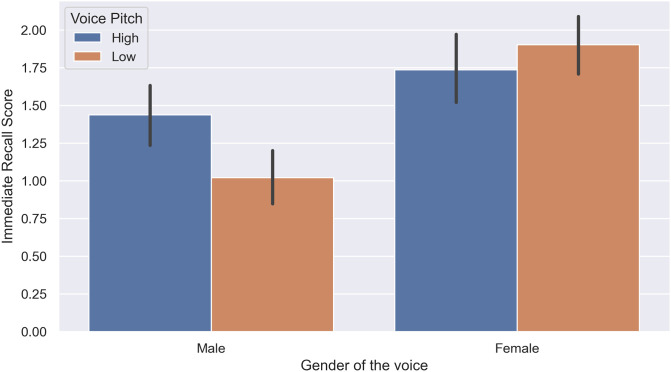
Interaction of voice gender and voice pitch in the immediate recall task (Experiment 1).

**Fig 3:**
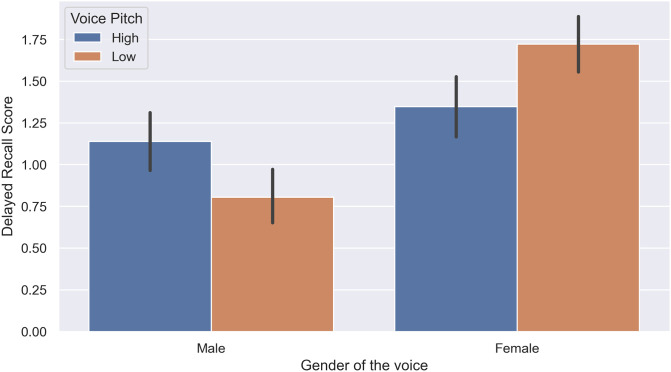
Interaction of voice gender and voice pitch in the delayed recall task (Experiment 1).

We observed a significant effect of the gender of the voice *F*(3, 140) = 24.99, *p* = .001, $\eta_{\text{p}}^{2}=.35$ on the memory performance of subjects. Furthermore, we observed a significant interaction between the gender of the voice and pitch of the voice in the immediate free recall task, *F*(1, 140) = 15.37, *p* = .062, $\eta_{\text{p}}^{2}=.1$, as shown in Fig 2, and delayed free recall task, *F*(1, 140) = 14.98, p = .001, $\eta_{\text{p}}^{2}=.09$, as demonstrated in Fig 3, but not in the recognition task, *F*(1, 140) = 2.96, *p* = .09, $\eta_{\text{p}}^{2}=.02$. Further post hoc analysis with Bonferroni adjustment revealed that when a male voice was used, a higher pitch resulted in significantly better memory performance in the recognition task (*p* = .007, 95%*CI* = [.20, 1.23]), immediate recall task (*p* = .001, 95%*CI* = [.25, .65]), and delayed recall task (*p* = .001, 95%*CI* = [.18, .58]). In contrast, a low-pitch female voice was remembered significantly better than a high-pitch female voice only in the delayed recall task (*p* = .004, 95%*CI* = [.1, .54]).

**Fig 4:**
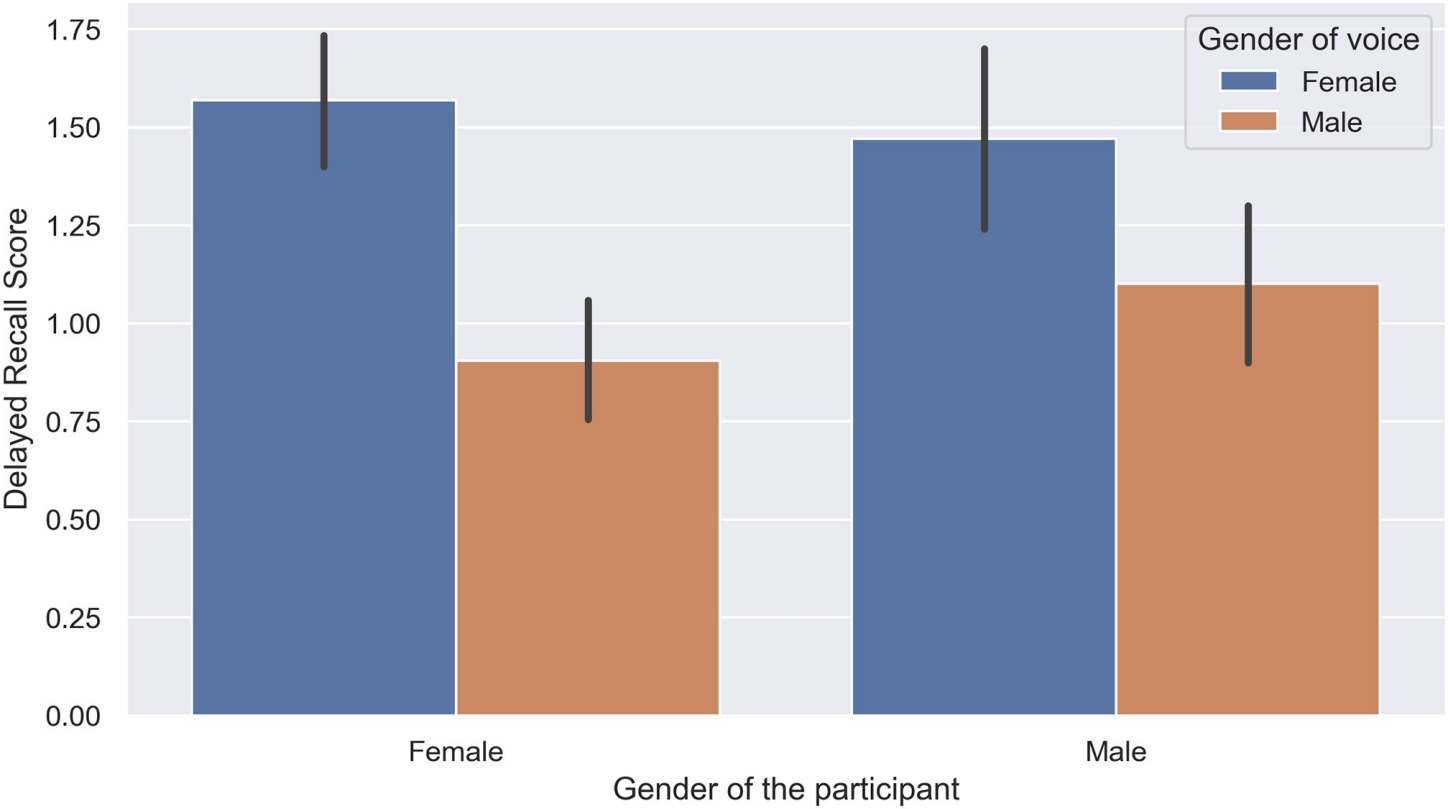
Interaction of participants’ gender and voice gender in the delayed recall task (Experiment 1).

In addition, we observed a significant interaction between the gender of the voice and the gender of the participant in the delayed recall task F (1, 140) = 4.99, p = .021, $\eta_{\text{p}}^{2}=.035$ shown in Fig 4. Post hoc analysis revealed that when a female voice was used, both male subjects (*p* = .002, 95%*CI* = [.15, .64]) and female subjects (*p* = .001, 95%*CI* = [.51, .91]) got a significantly higher score in the immediate recall task, and also after 5 minutes of delay. However, the presence of white noise did not affect the score of subjects significantly *F*(3, 140) = 1.51, *p* = .212, $\eta_{\text{p}}^{2}=.03$.

Eventually, the effect of working memory on the retrieval of presented information was significant, F (3, 137) = 5.73, p = .001, $\eta_{\text{p}}^{2}=.11$, which is in accordance with previous studies on this matter [32].

#### 3.1.5 Discussion

The aim of Experiment 1 was to determine the effects of the gender and pitch of the voice on the memory performance of subjects regarding the presented content, demonstrated in their score in word recognition, immediate word recall, and delayed word recall tasks. We evaluated these three different aspects of human memory, ranging from the least challenging one which requires less cognitive load (recognition) to the most challenging (delayed free recall), to have a wider view of potential interactions between the intended voice characteristics and the subject’s performance. Based on previous research, free and cued recall were more sensitive to experimental manipulations [33]; hence, they are better candidates for more delicate effects. This idea was confirmed in our experiment. While the words expressed by female voices were remembered better by both male and female subjects, we only observed this in the immediate recall and delayed recall tasks, but not in the word recognition task. The same pattern of results was true for the interaction between the gender and pitch of the voice.

Based on the acquired results, using a female voice had a significant positive effect on the memory retrieval of subjects of both genders. Our results in this experiment suggest a significantly better memory performance in human subjects when using a female voice, particularly when it had a lower pitch, and can justify the tendency of big tech companies to use an AI-generated female voice with a slightly lower pitch over a male voice for their virtual assistants.

Furthermore, we observed a significant effect of voice pitch on the memory of subjects regarding the presented content. Interestingly, while a higher pitch resulted in better memory of the presented content when using a male voice, a lower pitch resulted in better performance when using a female voice. This suggests a differential effect of the voice pitch when using different voice genders. In previous studies, a tendency in female subjects for a low-pitched male voice was observed and contributed to the attraction of masculinity in the male voice because of evolutionary mating preferences [34] in women. We failed to confirm this finding in our study. Even though previous studies have failed to show any significant differences in the speech comprehension of subjects when the voice is natural (human voice) versus when it is synthetic (computer-generated) [35], authors do not rule out the possibility that the acquired results in this experiment could be partly due to the use of synthetic voices. However, as the findings of this line of study are intended to be used in robotic experiments, the use of synthetic voices is the only available option. However, it is suggested that future studies consider comparing the differences when the talker uses a synthetic voice versus when a human voice is used in competing talker scenarios.

## 4 Experiment 2

The main contribution of Experiment 1 is that using a low-pitch female voice would result in significantly better memory performance in both male and female subjects; however, in Experiment 1, only one language (English) was presented to each participant. Therefore, it did not reveal how these factors would affect the performance of subjects in a bilingual setting when each subject hears two competing talkers at the same time and should only concentrate on the target voice (English) that he/she understands. To this end, another experiment was conducted to evaluate the interactions between voice gender and voice pitch in a competing talker scenario.

### 4.1 Methods

#### 4.1.1 Participants

A total of 249 subjects (124 female, 125 male) aged between 18 and 50 years (mean = 32.8, SD = 9.0) participated in this study. We used the Prolific online research platform to recruit the participants. Considering how monolingual and bilingual individuals perform differently in competitive talking scenarios on the neural and cognitive levels [36], participants were recruited from monolingual English-speaking people, currently residing in the United States of America, who were screened by Prolific not to have any hearing difficulties and had no understanding of the Russian language. Participants were asked to wear headphones during the task and attend the task in a quiet room. At the beginning of the experiment, participants were asked to adjust the volume of their computer using the same procedure mentioned in experiment 1.

#### 4.1.2 Design and procedure

A between-subjects experimental design was used to assess the speech comprehension of the subjects in a bilingual setting. The independent variables were the target language’s voice pitch (high pitch, low pitch), the background language’s voice pitch (high pitch, low pitch), and the background language’s voice gender (male and female). The dependent variables were the scores of the subjects in a speech comprehension test and their subjective experience of intelligibility, and their tendency to spend more time with the virtual robot in the future. After completing a written consent form and undergoing the backward digit span test as a measure of working memory, each participant was randomly assigned to one of the eight conditions shown in Table 2. Participants were then asked to listen to a short speech given by a tour guide robot introducing the facilities, working hours, and food options available in a summer resort where they were going to spend a weekend. As a cover story, each participant was told that they are listening to the speech given by the robot simultaneously with someone from another country.

After the speech was finished, participants were asked to answer six questions about the content of the presented speech. Furthermore, they were asked two questions that measured their subjective evaluation of the experience they had with the virtual agent. they could respond to these two questions by rating on a Likert scale ranging from 0 to 5. The first question was, ‘How easy was it for you to stay concentrated on the presented speech?’ and the Second question was “how could you rate your feelings toward the assistive robot as if she was going to be your assistive robot during your stay in such a resort?”. Participants could leave the experiment at any time by pressing the escape button twice on their keyboards.

#### 4.1.3 Materials

To increase the ecological validity of the findings in this experiment, we changed our task from a classic word-list memorization paradigm to a speech comprehension task design in which the to-be-retrieved information was presented in form of a speech presented by an assistant robot in an imaginary tour in a summer resort. Instead of manually changing the pitch of each voice in this experiment, we used AI characters with natural higher and lower pitches defined in Notevibes software. Because of the significantly poor performance of participants when using a male voice in Experiment 1 in this experiment, we only used the female voice for the target language (English). However, we retained voice gender as one of the factors in the background language (Russian) to evaluate the potential interactions between two different voice genders in the simultaneously presented languages. The frequency of each voice is presented in Table 1. All speech excerpts were checked for their LTAS using Praat software to make sure that they follow a similar long-term average spectrum.

**Table 1:** Frequency of each voice group generated for experiment 2.

Voice Characteristics			.
Gender	language	Pitch	Frequency
Female	English	Low Pitch	195 Hz
		High Pitch	227 Hz
Male	Russian	Low Pitch	90 Hz
		High Pitch	160 Hz
Female		Low Pitch	175 Hz
		High Pitch	208 Hz

Considering three independent factors (target voice pitch, background voice pitch, and background voice gender), each with two levels (high vs. low, male vs. female), we created a list of eight possible combinations of voices presented in Table 2. The SNR was set to -5 dB in all conditions in favor of the target language (English).

**Table 2:** Description of experiment conditions (Experiment 2).

.	Foreground language			Background language		
Conditions	Gender	Language	Pitch	Gender	Language	Pitch
1	Female	English	High Pitch	Female	Russian	High Pitch
2						Low Pitch
3				Male		High Pitch
4						Low Pitch
5			Low Pitch	Female		High Pitch
6						Low Pitch
7				Male		High Pitch
8						Low Pitch

#### 4.1.4 Results

A between-subjects experimental design was used to assess the speech comprehension of the subjects in a bilingual setting. The dependent variables were the scores of the subjects in the speech comprehension test, and their subjective evaluation of speech intelligibility, and comfortability while listening.

#### 4.1.5 Speech comprehension

We did not observe any significant effects of the target language’s voice pitch, F (1, 233) = .43, p = .51, $\eta_{\text{p}}^{2}=.002$, background language’s voice pitch, F (1, 233) = .7, p = .4, $\eta_{\text{p}}^{2}=.003$, and background language’s voice gender, F (1, 233) = .21, p = .64, $\eta_{\text{p}}^{2}=.001$ on the speech comprehension of subjects. The effect of working memory on the scores of subjects in the speech comprehension task was significant, F (1, 233) = 18.31, p = .001, $\eta_{\text{p}}^{2}=.07$. We did not observe a significant interaction between participants’ gender, target speaker’s voice pitch, and background speaker’s gender, F (1, 233) = .53, p = .47, $\eta_{\text{p}}^{2}=.002$.

#### 4.1.6 Subjective experience

We found a significant relationship between the gender of the participants and their subjective experience during the experiment demonstrated in their tendency to spend more time with the assistive robot, using the chi-square test of independence X2 (2, N = 249) = 7.63, p = .022. Women were more likely to evaluate their experiences positively (48.4%) than men (32.8%). We could not observe any other significant relationships between the evaluated factors and participants’ subjective experience or intelligibility of the presented content.

#### 4.1.7 Discussion

Considering the findings of this experiment, neither the gender nor pitch of the presented speech seemed to have a significant effect on participants’ speech comprehension in a bilingual setting. Based on one of the principles of perceptual psychology, namely the contrast effect, the presence of a stimulus in the background with a significantly different characteristic simultaneous to the target stimulus results in enhanced or reduced attention to the target stimulus [37]. The same principle has been shown before in the auditory domain as well when presenting two voices with significantly different fundamental frequencies resulting in improved signal segregation for the target voice [38]. We expected to observe a similar pattern in the simultaneously presented bilingual speech. In such a case, we would expect a higher score when, for example, a high-pitched female voice was accompanied by a low-pitch male voice in the background. The rationale behind this expectation was that the contrast between the two messages would make it easier for participants to stay focused on the target message while ignoring the background content. However, we could not confirm this pattern of results in this experiment.

One possible reason behind our failed attempt to observe any significant interaction between the target voice’s pitch and the background voice’s pitch on the performance of subjects might be due to using only one task (speech comprehension) to measure such an effect. The rationale of our decision to limit the task to the current one was first, to keep the task short and prevent fatigue in subjects; and second, to resemble the natural context where the result of this line of studies will be used “A simultaneously-speaking bilingual robot”. Therefore, we tested the comprehension of the subjects by asking them about the main ideas, and details of the presented robot speech instead of using the classic word, and sentence recall tasks.

## 5 Conclusion

In this study, which consisted of two experiments, we investigated whether adjusting the pitch, and the gender of the robot’s voice would improve the intelligibility of presented speech information in the presence of non-verbal (white noise) and verbal (speech in a foreign language) noise. It has been well documented in previous studies that the presence of any type of noise deteriorates the performance of subjects in auditory tasks [39]. This effect is stronger when the background noise is human speech, even if it is in a foreign language [40]. In Experiment 1, we reached some solid conclusions on the necessity of using a female voice to enhance information retrieval in a non-verbal noisy condition. In the second experiment, we kept the gender of the target voice constant and evaluated the effect of the target voice’s pitch, the background voice’s pitch, and the background voice’s gender on the speech comprehension of participants. However, we failed to observe a significant effect of voice pitch on its own, on the speech comprehension score of subjects. This is contrary to the previous works that suggest a difference between the pitch of the target voice, and the background voice can have a facilitatory effect on the speech comprehension of the subjects. And confirms the more recent works that suggest the effect of voice pitch on the cognitive performance of subjects to be negligible.

Considering the limitations and lack of control in an online experiment setup and the possibility of performing on-site experimentation owing to the progress of the COVID-19 vaccination in Japan, in a future study, we plan to repeat the second experiment in a more controlled manner using English-speaking and Japanese-speaking subjects, and physical robots to determine whether the results will differ from the current set of experiments which used auditory information only. Another limitation of the current study is that it emphasizes the memory performance of participants as an indirect indication of their tendency toward a specific voice characteristic, which might not be the most accurate method to evaluate such a matter. Although we included a subjective evaluation in Experiment 2 to have a direct evaluation of the subjects, still in a future study, more elaborate methods like using subjects’ physiological data, and evaluation of subconscious choice biases are considered.

The takeaway of this study could be summarized by the fact that, while voice gender has a distinct effect on the memory performance of subjects in a noisy situation, voice pitch fails to produce the same strength of effect on its own. Hence, in the acoustic designing of a simultaneously speaking bilingual robot or a broader range of multilingual public address systems serving in noisy conditions, using a female voice is recommended for better intelligibility and memory.
